# *GlPP2C1* Silencing Increases the Content of *Ganoderma* *lingzhi* Polysaccharide (GL-PS) and Enhances Slt2 Phosphorylation

**DOI:** 10.3390/jof8090949

**Published:** 2022-09-10

**Authors:** Zi Wang, Ju-Hong Chen, Ling-Shuai Wang, Juan Ding, Ming-Wen Zhao, Rui Liu

**Affiliations:** Key Laboratory of Microbiological Engineering of Agricultural Environment, Ministry of Agriculture, Department of Microbiology, College of Life Sciences, Nanjing Agricultural University, Nanjing 210095, China

**Keywords:** PP2C, *Ganoderma lingzhi*, GL-PS, Slt2

## Abstract

Polysaccharides have attracted much attention in the food industry due to their diverse biological activities. To date, research on the mechanism of polysaccharide synthesis has mainly focused on the role of crucial enzymes in the polysaccharide synthesis pathway, but other genes that regulate polysaccharide synthesis have not been well explored. In this study, the *GlPP2C1* gene, encoding a phosphoprotein type 2C phosphatase, was cloned, and PP2C-silenced strains (PP2C1i-1 and PP2C1i-3) were screened. Measurements of the polysaccharide content and cell wall tolerance revealed that *GlPP2C1* silencing increased the polysaccharide content and enhanced cell wall resistance in *Ganoderma lingzhi.* The contents of intracellular polysaccharides (IPS), extracellular polysaccharides (EPS) and β-1,3-D-glucan in PP2C-silenced strains were increased by 25%, 33% and 36%, respectively, compared with those in the WT strains and strains transformed with an empty vector. Further mechanistic studies showed that *GlPP2C1* silencing increased the content of *Ganoderma lingzhi* polysaccharides (GL-PS) through Slt2. In summary, this study revealed the mechanism through which protein phosphatase regulates GL-PS biosynthesis for the first time.

## 1. Introduction

Polysaccharides are one of the fundamental substances of life and have complex structures and wide distribution. Polysaccharides are essential biological macromolecules that usually exist in the form of intracellular polysaccharides (IPSs) and extracellular polysaccharides (EPSs). In addition to the essential functions of structural polysaccharides in maintaining organisms, some medicinal fungal polysaccharides have been widely reported to have a variety of immune regulatory functions. For instance, the polysaccharides in *Agaricus blazei Murrill* have the potential to prevent or treat inflammatory diseases, such as cancer, diabetes, and atherosclerosis [1]. Polysaccharides from *Cordyceps cicadae* spores can significantly increase the splenic and thymic indices, enhance the phagocytic activity of macrophages, improve the cytotoxicity of natural killer (NK) cells, and regulate the secretion of cytokines and immunoglobulins to exert an improving effect in cyclophosphamide-induced immunosuppressive mice [2]. Polysaccharides from *Hericium erinaceus* have strong vitamin antioxidant activity and protective effects on carbon tetrachloride-induced liver injury in mice [3]. Due to these biological activities, polysaccharides have attracted much attention in the field of functional foods.

*Ganoderma lingzhi,* a traditional medicinal mushroom, has been used in Asia for thousands of years to treat various human diseases, such as cancer, inflammation, chronic hepatitis, heart disease, and hypertension [4,5,6,7]. These activities are due to a critical bioactive compound, *G. lingzhi* polysaccharide (GL-PS). A large number of studies have shown that GL-PS has antioxidation, antibacterial, immune regulation, antitumor, blood lipid-lowering, blood sugar-lowering, and liver protection functions and has been widely used in medicine and health foods [8,9]. However, most recent studies have focused on the isolation, purification and pharmacology of GL-PS, but the molecular mechanism of GL-PS biosynthesis has rarely been examined.

GL-PS is mainly composed of EPSs and IPSs. EPSs are secreted by cells to the outside of the cell. IPSs are mainly found in cells. At present, the research on GL-PS has mainly focused on two aspects. Firstly, different carbon sources, nitrogen sources, ions, medium pH values, and culture temperatures can affect polysaccharide production [10]. Moreover, the growth of mycelium and the production of EPSs are affected when the pH of the fermentation is controlled by the two-step fermentation method [11]. Secondly, genes related to GL-PS biosynthesis have been studied, and the genes encoding key enzymes involved in polysaccharide synthesis have been isolated and identified. For example, existing studies have found that crucial enzyme genes, such as α-phosphoglucomutase (PGM) and UDP-Glc pyrophosphorylase (UGP), that are crucial in the polysaccharide synthesis pathway can affect GL-PS synthesis [12,13]. In addition, some genes regulating GL-PS synthesis have been discovered recently. Heme oxygenase (HO), an enzyme that catalyzes the decomposition of heme into bilirubin and produces iron and carbon monoxide simultaneously, has been found to positively regulate GL-PS synthesis [14]. *GlbHLH*, a basic helix-loop-helix (bHLH) transcription factor in *G. lingzhi,* was also found to positively regulate polysaccharide biosynthesis [15]. These genes are not vital enzyme genes in the polysaccharide synthesis pathway but exert significant regulatory effects on GL-PS biosynthesis. Few studies have investigated these genes, and further exploration of these genes could help provide genetic resources for improving GL-PS content, which will play an essential role in the further development of GL-PS.

When the cell wall is stressed by the external environment, the cell wall integrity (CWI) signaling pathway is initiated. The output of this pathway is primarily driven by the activity of MAPK Slt2 [16]. Activation of the CWI pathway stimulates the Slt2-specific MAPKKs Mkk1 and Mkk2, which phosphorylate threonine (T190) and tyrosine (Y192) residues within the T-E-Y motif of the Slt2 activation loop [17]. Recent studies of Slt2 found that the T195 residue is also critical for Slt2 function [18]. However, Slt2 is not always in a phosphorylated state. The serine/threonine phosphatase Ptc1 affects cell wall integrity Slt2 MAPK by dephosphorylating Mkk1, which encodes for a MAPK kinase (MAPKK) known to activate Slt2 [19]. In addition, protein phosphatase can directly act on Slt2 to affect its phosphorylation state, as has been observed with the tyrosine phosphatases Ptp2 and Ptp3 and the dual specificity phosphatases (DSPs) Msg5 and Sdp1 [20]. However, the protein phosphatase affecting Slt2 in *G. lingzhi* has not been explored.

Type 2C protein phosphatases (PP2Cs) are PPM family phosphatases because their activity is dependent on metal ions [21]. Research on the role of PP2C enzymes in fungi has mainly focused on *Saccharomyces cerevisiae*, among which there are seven PP2C species (PTC1-PTC7), and the best characterized PP2C is PTC1. Studies in recent years have shown that PTC1 not only negatively regulates the CWI signaling pathway but is also involved in tRNA splicing, sporulation, lithium tolerance, vacuolar endoplasmic reticulum dynamics and during cell division [22]. In addition, Ptc2 and Ptc3 are postulated to be protein phosphatases that dephosphorylate cyclin-dependent kinases and are needed for checkpoint inactivation following DNA double-strand breaks. Cells lacking Ptc6 are sensitive to zinc ions, and somewhat tolerant to cell-wall damaging agents and to Li^+^. Ptc6 is needed for proper rapamycin-induced downregulation of the genes encoding for ribosomal and rRNA processing proteins in S. cerevisiae [23,24]. In conclusion, the published studies clearly show that PP2C plays a vital role in the response and physiological function of various environmental stresses. However, to the best of our knowledge, the function of PP2C in basidiomycetes has not been reported.

In this study, the *GlPP2C1* gene was identified in *G.* *lingzhi*. To verify the role of *GlPP2C1* in physiological processes, two independent PP2C1-silenced strains (PP2C1i-1 and PP2C1i-3) were generated using RNA interference (RNAi). Our results showed that *GlPP2C1* silencing exerted a positive regulatory effect on polysaccharide synthesis and cell wall integrity in *G.* *lingzhi*. Moreover, our results also verified that *GlPP2C1* silencing increased polysaccharide biosynthesis mediated by Slt2 in response to cell wall integrity. In conclusion, our study provides the first demonstration that *GlPP2C1* silencing exerts a positive regulatory effect on polysaccharide biosynthesis and lays the foundation for further elucidation of the mechanism of polysaccharide biosynthesis.

## 2. Materials and Methods

### 2.1. Strains and Culture Conditions

The wild-type (WT) strain was collected in 1996 and deposited in the Agricultural Culture Collection of China under the number ACCC53264. All strains were cultured at 28 °C on complete yeast medium (CYM) for hyphal growth tests, as needed [25]. The Slt2-silencing strains (Slt2i-5 and Slt2i-9) were constructed previously [26]. In short, Slt2i-5 and Slt2i-9 strains were constructed and screened using RNAi technology. The transcript levels in the Slt2-silenced strains decreased to 10% of the WT level. CYM medium was used for the detection of GL-PS biosynthesis, biomass and stress resistance. For liquid fermentation, the mycelial blocks were inoculated in CYM and placed on a rotary shaker incubator at 150 rpm and 28 °C for 7 days.

### 2.2. Transformant Construction

The silenced transformants were constructed using an RNAi-mediated gene silencing strategy. The fungal RNAi vector pAN7-ura3-dual was used for the construction of PP2C1-silenced strains of *G. lingzhi,* which were transformed by electroporation as previously described [27]. With *G. lingzhi* cDNA as a template, using rTaq enzyme (Takara, Dalian, China), along with KpnI and SpeI (Takara, Dalian, China) as restriction sites, the PP2C coding region fragment was amplified. The primers are shown in Table S1. The amplified DNA fragment was ligated into the pAN7-ura3-dual vector with SpeI and KpnI as restriction sites and transformed into *G. lingzhi*. Silenced nucleotide sequences are provided in the Supplementary Materials. The specificity of the silenced fragments was demonstrated by multiple sequence alignment of the five PP2Cs of *G. lingzhi* (Figure S1). The transformants were first selected on CYM medium containing 100 μg mL^−1^ hygromycin B and then selected on CYM with 600 mg mL^−1^ 5-fluoroorotic acid (Sangon Biotech, Shanghai, China) to screen the silenced strains [28]. The PP2C-silenced strains were called PP2C1i-1 and PP2C1i-3. The empty vector control was named CK.

### 2.3. Gene Expression Analysis

Total RNA was extracted from 100 mg of hyphae using RNAiso Plus (TaKaRa, Dalian, China). A 5× All-In-One RT MasterMix kit (with AccuRT Genomic DNA Removal Kit, ABM, Richmond, BC, Canada) was used to obtain cDNA for the analysis of gene expression. RT-PCR was performed using cDNA as a template, various primers (Table S1) and SYBR Green I Mix. A quantitative RT-qPCR assay was then performed using Eppendorf Mastercycler Ep Realplex 2.2 software (Eppendorf, Hamburg, Germany). The transcription levels of gene-specific mRNAs were analyzed using 18S rRNA as the housekeeping gene [29]. All the primers used in this experiment are shown in Table S1. The 2^−ΔΔCT^ method was used to determine the relative expression levels of the genes [30].

### 2.4. Western Blotting

Total protein (25 μg) from the samples was separated on a 12% (wt·vol^−^^1^) SDS–PAGE gel, transferred to polyvinylidene difluoride membranes (Bio-Rad, Hercules, CA, USA) and incubated with anti-phospho Ser/Thr (1:1000, Abcam, Cat# ab117253), anti-pERK (1:1000, Cell Signaling Technology, Cat# 4376) or anti-ERK (1:1000, Cell Signaling Technology, Cat# 9102) antibodies and then with a secondary horseradish peroxidase (HRP)-conjugated goat anti-rabbit IgG antibody. Finally, blots were developed using the ECL Western blotting detection system (Amersham Bioscience, Uppsala, Sweden). A density analysis of each group was performed using ImageJ v1.8.0 software.

### 2.5. Growth Rate Experiments under Different Stresses

The WT, CK and PP2C-silenced strains were cultured at 28 °C on complete yeast medium (CYM) or cell wall stressors (0.05% sodium dodecyl sulfate (SDS), 1 mg mL^−1^ calcofluor white (CFW) or 4 mg mL^−^^1^ Congo red (CR)) based on a previous method [31]. The relative growth rate was calculated using the following formula: relative growth rate (%) = (growth area on stress media/growth area on CYM) × 100.

### 2.6. Quantification of β-1,3-D-glucan

The β-1,3-D-glucan content was determined by aniline blue staining according to previously described methods [32]. In short, fluorescence readings were obtained using a Tecan Infinite M200 fluorometer with excitation at 405 nm and emission at 460 nm. The absorbance was measured at 650 nm with a Vis spectrophotometer (Shimadzu Corporation, Kyoto, Japan). For normalization of the values, a standard curve was created using curdlan, a β-1,3-D-glucan analog (Sigma, St. Louis, MO, USA). The values are expressed as the percentage changes in relative fluorescence units per milligram of mycelial tissue using WT as the control.

### 2.7. Determination of EPS and IPS

EPS and IPS were analyzed according to previously described methods [33]. Hyphae were removed by centrifugation of liquid shaken cultures grown for 7 days at 28 °C and 150 rpm in mushroom complete medium. Crude EPS was precipitated from the supernatant using the following method: In short, after adding four volumes of 95% (v·v^−1^) ethanol, the supernatant was stirred vigorously and then incubated overnight at 4 °C. After centrifugation, insoluble fractions were suspended at 60 °C for 1 h using 1 M NaOH. The supernatant was collected by centrifugation, and the EPS content was measured using the phenol-sulfuric acid method. Hyphae were collected by centrifugation of liquid shaken cultures grown for 7 days at 28 °C and 150 rpm in mushroom complete medium and were used for IPS analysis. The constant dry weight was measured after drying at 60 °C for 28 h, and IPS then was extracted with 1 M NaOH at 60 °C (1 h). The supernatant was collected by centrifugation, and the IPS content was measured using the phenol-sulfuric acid method, with glucose as a standard.

### 2.8. Quantification of Hyphal Branches

A Nikon Eclipse Ti-S microscope (Nikon, Tokyo, Japan) was used to analyze the hyphae of WT, CK and PP2C-silenced strains as described previously. Fluorescent Brightener 28 (Sigma, St. Louis, MO, USA) was used for staining vegetative hyphae, and related staining and fluorescence detection methods were performed as described previously [34]. The length between hyphal branches was quantified using a previously described method [35].

### 2.9. Statistical Analysis

The statistical analyses were performed using GraphPad Prism 8. All experimental data shown in this article were obtained from three independent samples to ensure that the trends and relationships observed in the cultures were reproducible. The error bars indicate the standard deviation (SDs) from the means of triplicates. The differences in mean values between groups were analyzed by one-way or two-way analysis of variance (ANOVA) using GraphPad Prism. * *p* < 0.05, ** *p* < 0.01, *** *p* < 0.001 and **** *p* < 0.0001 are the levels of statistical significance. NS is not significant.

## 3. Results

### 3.1. Cloning and Sequence Analysis of the GlPP2C1 Gene

PP2C of *Dichomitus squalens* was used in a Blast search against the *Ganoderma lingzhi* genome database (http://www.herbalgenomics.org/galu/; URL accessed date: 2 January 2021) to identify PP2C homologs in *G. lingzhi*. A total of five PP2Cs were found in *G. lingzhi*. The five PP2C nucleotide sequences were aligned, and the identity was 43.85% (Figure S1). A close homolog (GL22901-R1) was identified in the database and named *GlPP2C1*. The length of the *GlPP2C1* cDNA was 1545 bp, and predictions indicated that this cDNA encodes a protein of 515 amino acids with a molecular mass of 56.11 kDa (http://www.novopro.cn/tools/; URL accessed date: 2 January 2021). Analysis of the predicted amino acid sequence of *GlPP2C1* identified one conserved domain (http://smart.embl-heidelberg.de/; URL accessed date: 2 January 2021), the PP2C_SIG domain, which can be easily detected in multiple sequence alignments of PP2C-like phosphatases (Figure S2A). We compared the sequence of GL22901-R1 (OP251201) with those of PP2Cs of other basidiomycetes and found 61.66%, 47.40% and 49.52% similarity with *D. squalens* (TBU29298.1), *Lentinus tigrinus* (*RPD55288.1*) and *Polyporus brumalis* (RDX47371.1), respectively (Figure S2B). This gene contained the PP2C domain and was highly similar to the PP2C genes in other species, which implies that it was *GlPP2C1* in *G. lingzhi*. We also performed a multiple sequence alignment of Gl22901-R1 with *D. squalens* (TBU29298.1), *Lentinus tigrinus* (RPD55288.1) and *S. cerevisiae* PP2Cs (PTC1-PTC7) and found 25.67% identity (PTC1 NC_001136.10, PTC2 NC_001137.3, PTC3 NC_001134.8, PTC4 NC_001134.8, PTC5 NC_001147.6, PTC6 NC_001135.5, PTC7 NC_001140.6) (Figure S3). However, the identity of Gl22901-R1 with basidiomycetes and *S. cerevisiae* was 60% and 25%, respectively, and the identity of Gl22901-R1 with the basidiomycetes in the multiple sequence alignment was markedly higher than that with *S. cerevisiae*. This result indicated that *GlPP2C1* of *G. lingzhi* is conserved in basidiomycetes.

### 3.2. Construction of GlPP2C1 RNAi Strains in G. lingzhi

**Figure 1:**
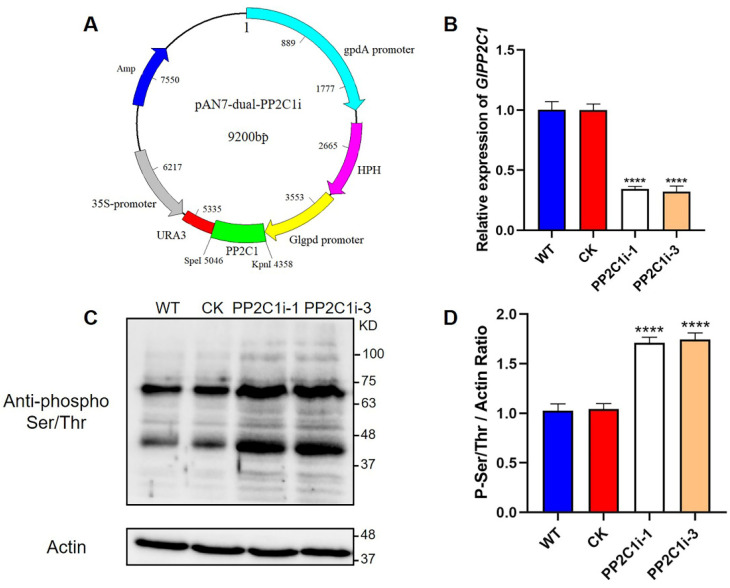
Detection of the *GlPP2C1* transcript levels and the phosphorylation of Ser/Thr in PP2C-silenced strains. (**A**) Plasmid map of the PP2C silenced gene. (**B**) qRT–PCR analysis of the expression of *GlPP2C1* in the tested strains. (**C**) Western blotting analysis of the effectiveness of PP2C-silenced strains with phospho-Ser/Thr antibodies. The actin protein level was used as a loading control. (**D**) The relative intensity of the two prominent phospho-Ser/Thr bands was determined using ImageJ software. The data are presented as the means ± standard deviations (SDs) from three independent experiments (**** *p* < 0.0001 by one-way ANOVA).

To explore the function of *GlPP2C1* in *G. lingzhi,* a PP2C-silencing vector with a dual promoter system was used, as described previously. The dual promoter refers to the GPD promoter and the 35S promoter, and due to its high silencing efficiency, this dual promoter is widely used in fungi [23]. The PP2C-silencing vector is shown in Figure 1A, and the pAN7-ura3-dual vector was used as an empty vector control. A structure of the vector constructed for silencing the expression of *GlPP2C1* is shown in Figure S4. Forty-one candidate strains were obtained after hygromycin screening, and real-time quantitative polymerase chain reaction (RT–qPCR) analysis was performed to detect the transcription level of *GlPP2C1* in 41 screened candidate strains with possible gene silencing. Two strains (PP2C1i-1 and PP2C1i-3), with the highest silencing efficiency among the 41 candidate strains screened for gene silencing, were selected for follow-up experiments. The expression of the *GlPP2C1* gene in the PP2C1i-1 and PP2C1i-3 strains was decreased by approximately 65 and 70%, respectively, compared with the WT level (Figure 1B).

PP2Cs contain a conserved Ser/Thr protein domain and are members of the protein phosphatase family. To confirm that the decreased level of PP2Cs in the PP2C1i-1 and PP2C1i-3 mutants affected the phosphorylation status of phosphoproteins, protein phosphorylation was assessed using anti-phospho Ser/Thr antibodies (Figure 1C). The immunoblotting results showed that the signal intensity of phospho Ser/Thr in the PP2C1i-1 and PP2C1i-3 strains was increased by 71 and 74%, respectively, compared with that in the WT (Figure 1D). These results demonstrated that the PP2C1i-1 and PP2C1i-3 strains screened are effective and can be used for further physiological research.

### 3.3. GlPP2C1 Contributed to GL-PS Biosynthesis in G. lingzhi

**Figure 2:**
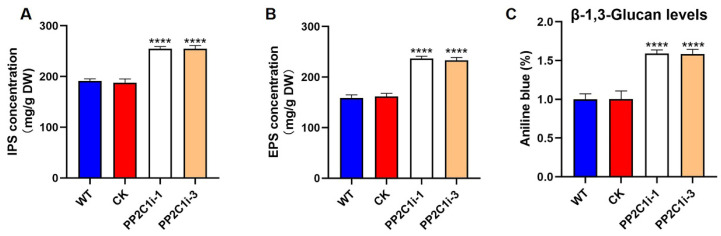
Effect of *GlPP2C1* on GL-PS biosynthesis. (**A**) IPS content in WT, CK and PP2C-silenced strains. (**B**) EPS content in WT, CK and PP2C-silenced strains. (**C**) β-1,3-D-glucan content in WT, CK and PP2C-silenced strains. The data are presented as the means ± SDs from three independent experiments (**** *p* < 0.0001 by one-way ANOVA).

Polysaccharides are important in *G. lingzhi.* Therefore, we tested the contents of intracellular polysaccharides (IPSs), extracellular polysaccharides (EPSs) and β-1,3-D-glucan. The IPS contents in both the PP2C1i-1 and PP2C1i-3 strains were 25% higher (Figure 2A) than in the WT and CK strains. In addition, the EPS contents in the PP2C1i-1 and PP2C1i-3 strains were 33% and 32% higher, respectively (Figure 2B), than in the WT and CK strains. Compared with those in the WT and CK strains, the contents of β-1,3-D-glucan in the PP2C1i-1 and PP2C1i-3 strains were increased by 37% and 36%, respectively (Figure 2C). These results showed that *GlPP2C1* silencing played a role in GL-PS biosynthesis and had a positive effect.

### 3.4. GlPP2C1 Gene Silencing Increases Cell Wall Stress Tolerance in G. lingzhi

**Figure 3:**
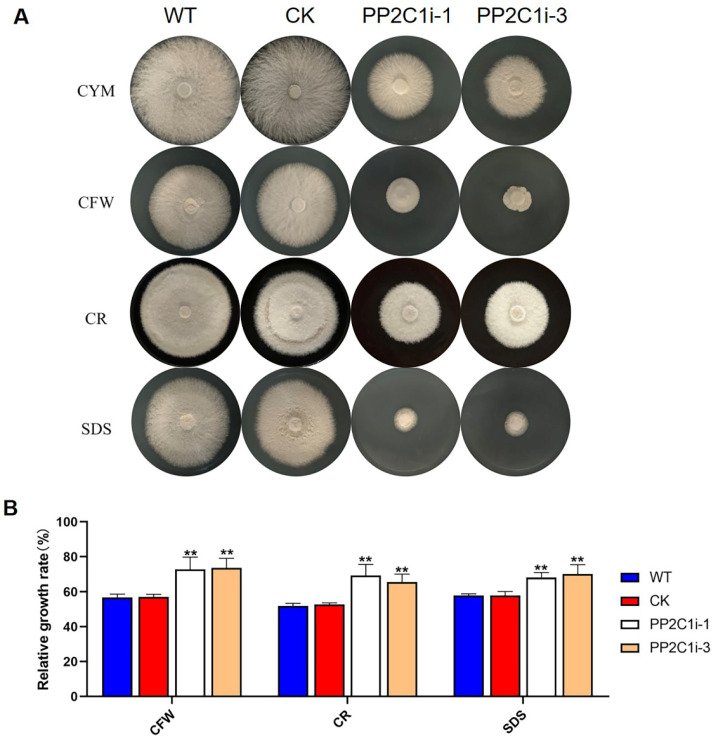
Sensitivity of PP2C-silenced strains to cell wall-disrupting compounds. (**A**) The PP2C-silenced, WT and CK strains were cultured on CYM plates under different cell wall stresses. (**B**) Relative growth rates of the tested strains under cell wall stress in the PP2C-silenced, WT and CK strains. The relative growth rates (%) of the different strains in the presence of the indicated stressors were calculated as the colony area on stressor-containing medium versus the colony area on CYM. The data are presented as the means ± SDs from three independent experiments (** p < 0.01 by one-way ANOVA).

*G. lingzhi* may experience various abiotic stresses during its growth and development. GL-PS plays an important role in resisting abiotic stress. Therefore, we examined the relative growth rates in the WT, CK, PP2C1i-1 and PP2C1i-3 strains under cell wall stress (Figure 3A). The measured relative growth rates are presented in Figure 3B. In CYM medium, the PP2C1i-1 and PP2C1i-3 strains grew 50% slower than the WT and CK strains. Under CFW treatment, the relative growth rates of the WT and CK strains were 57%, whereas the relative growth rates of the PP2C1i-1 and PP2C1i-3 strains were 73% and 74%, respectively. The relative growth rate after PP2C silencing was significantly higher than that of the WT and CK strains. This finding suggested that the silencing of *GlPP2C1* increased the tolerance to CFW (Figure 3B). Similarly, after CR stress, the relative growth rates of the WT and CK strains were 52%. However, the relative growth rates of the PP2C1i-1 and PP2C1i-3 strains were 66% and 70%, respectively. Under SDS treatment, the PP2C1i-1 and PP2C1i-3 strains showed growth rates of 69% and 70%, respectively, which were clearly higher than those of the WT and CK strains. As a significant indicator of the mycelial growth process of filamentous fungi, the hyphal branching phenotype upon growth on solid plates was examined (Figure S5A). According to the observation, the distance between the aerial hyphal branches was shortened in the PP2C1i-1 and PP2C1i-3 strains and decreased by approximately 45% compared with that in the WT and CK strains (Figure S5B). In addition, to further illustrate the effects of PP2C proteins on the growth of *G. lingzhi*, we analyzed the biomass of all the strains. Notably, the dry weight of the PP2C1i-1 and PP2C1i-3 strains was significantly reduced by approximately 60% compared with that of the CK and WT strains (Figure S5C). In conclusion, the above results indicated that *GlPP2C1* silencing could increase the relative growth rate of *G. lingzhi* under cell wall stress, shorten mycelial bifurcation and reduce biomass.

### 3.5. GlPP2C1 Gene Silencing Increases Slt2 Phosphorylation

**Figure 4:**
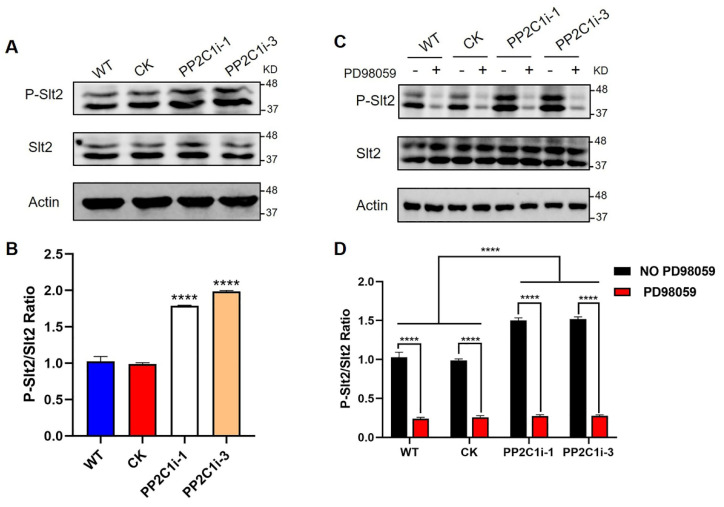
PP2C dephosphorylation of Slt2 in vivo. (**A**) The phosphorylation level of Slt2 in WT, CK and PP2C-silenced strains was detected with anti-pERK and anti-ERK antibodies. (**B**) P-Slt2/Slt2 ratio from panel A. (**C**) The phosphorylation level of Slt2 was detected with anti-pERK and anti-ERK antibodies after the addition of 20 μM PD98059 to the WT, CK and PP2C-silenced strains. (**D**) P-Slt2/Slt2 ratio from panel C. The data are presented as the means ± SDs from three independent experiments (**** *p* < 0.0001 by one-way ANOVA or two-way ANOVA).

Slt2 is a vital protein kinase in the cell wall stress pathway, and its phosphorylation level represents its activation state. We then detected the phosphorylation level of Slt2 in the WT, CK, PP2C1i-1 and PP2C1i-3 strains (Figure 4A). The immunoblotting results showed that the phosphorylation levels of Slt2 in the PP2C1i-1 and PP2C1i-3 strains were 1.7- and 1.9-fold higher, respectively, than in the WT and CK strains (Figure 4B). In addition, to further confirm the regulatory effect of PP2C on Slt2, we added different concentrations of PD98059, an inhibitor working upstream of Slt2 in the MAP kinase pathway and thereby blocks the phosphorylation cascade that activates Slt2 by phosphorylation, to the WT strain (Figure S6A). Western blot analysis also showed that the addition of different concentrations of inhibitors significantly decreased the phosphorylation level of Slt2 compared with that in the untreated WT strain (Figure S6B). However, when the inhibitor concentration was 20 μM, the phosphorylation level of Slt2 did not decrease significantly. Subsequently, we added PD98059 at a final concentration of 20 μM to the WT, CK, PP2C1i-1 and PP2C1i-3 strains (Figure 4C). Western blot analysis showed that the phosphorylation level of Slt2 in the strains with PD98059 was significantly lower than that in the strains without PD98059 (Figure 4D). The above results showed that *GlPP2C1* gene silencing increases Slt2 phosphorylation.

### 3.6. GlPP2C1 Silencing Positively Regulates Slt2-Mediated GL-PS Biosynthesis in G. lingzhi

**Figure 5:**
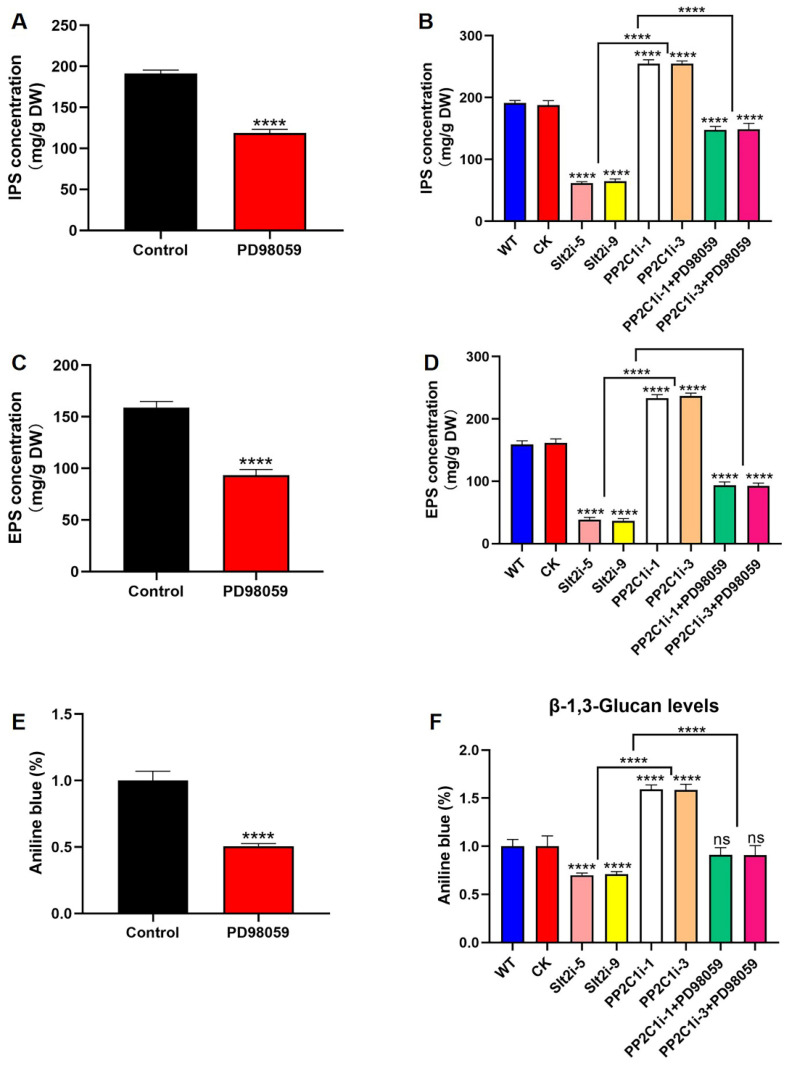
GL-PS content. (**A**) IPS contents in WT strains treated or not treated with 20 μM PD98059. (**B**) IPS contents in WT, CK, Slt2-silenced and PP2C-silenced strains treated with 20 μM PD98059. (**C**) EPS contents in WT strains treated or not treated with 20 μM PD98059. (**D**) EPS contents in WT, CK, Slt2-silenced and PP2C-silenced strains treated with 20 μM PD98059. (**E**) β-1,3-D-glucan content in WT strains treated or not treated with 20 μM PD98059. (**F**) β-1,3-D-glucan content in WT, CK, Slt2-silenced and PP2C-silenced strains treated with 20 μM PD98059. The data are presented as the means ± SDs from three independent experiments (**** *p* < 0.0001 by one-way ANOVA, ns, not significant).

To investigate the role of Slt2 in GL-PS biosynthesis, we added 20 μM PD98059 to the WT strain. The results showed that the inhibitor-added strains had IPSs, EPSs and β-1,3-D-glucan reduced by 38%, 41% and 50%, respectively, compared with the control level (Figure 5A,C,E). Moreover, the IPS contents of the Slt2i-5 and Slt2i-9 strains were 68% and 65% lower than in the WT and CK strains, respectively. The IPS contents of both the PP2C1i-1 and PP2C1i-3 strains treated with 20 μM PD98059 were reduced by 42% compared with the contents of both the untreated PP2C1i-1 and PP2C1i-3 strains (Figure 5B). Consistently, the EPS contents of the Slt2i-5 and Slt2i-9 strains were 76% and 77% lower than in the WT and CK strains, respectively. However, the EPS contents of both PP2C1i-1 and PP2C1i-3 treated with 20 μM PD98059 were reduced by 60% compared with the untreated PP2C1i-1 and PP2C1i-3 strains (Figure 5D). Furthermore, we examined β-1,3-D-glucan content in the WT, CK, Slt2i-5, Slt2i-9, PP2C1i-1, PP2C1i-3 and PP2C-silenced strains treated with 20 μM PD98059. Compared with the WT and CK strains, the β-1,3-D-glucan contents of the Slt2i-5 and Slt2i-9 strains were reduced by 31% and 29%, respectively. However, the addition of 20 μM PD98059 to the PP2C1i-1 and PP2C1i-3 strains reduced the contents of β-1,3-D-glucan by 42% and 43%, respectively, compared with the contents in the strains that were not treated with PD98059 (Figure 5F). In conclusion, Slt2 clearly functions as a positive regulator in GL-PS biosynthesis. In contrast, PP2C acts as a negative regulator of GL-PS biosynthesis mediated by Slt2.

### 3.7. PP2C Was Involved in the Cell Wall Integrity Pathway Mediated by Slt2 in G. lingzhi

**Figure 6:**
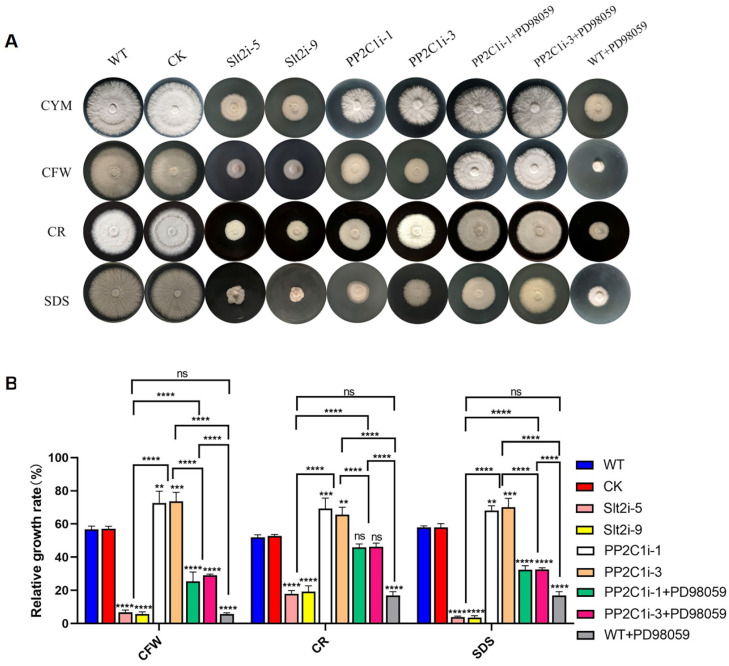
Sensitivity of different strains to cell wall-disrupting compounds. (**A**) The PP2C-silenced, WT, CK, Slt2-silenced and PP2C-silenced strains treated with 20 μM PD98059 were cultured on CYM plates under different cell wall stresses. (**B**) Relative growth rates of the PP2C-silenced, WT, CK, Slt2-silenced and PP2C-silenced strains under cell wall stress in the treated with 20 μM PD98059. The relative growth rates (%) of the different strains in the presence of the indicated stressors were calculated as the colony area on stressor-containing medium versus the colony areas on CYM. The data are presented as the mean ± SDs from three independent experiments (** p < 0.01, *** p < 0.001 and **** p < 0.0001 by one-way ANOVA, ns, not significant).

The above results confirmed that *GlPP2C1* gene silencing increases Slt2 phosphorylation, whereas Slt2 positively regulates GL-PS biosynthesis. Subsequently, we examined the relative growth rates of the WT, CK, Slt2i-5, Slt2i-9, PP2C1i-1, PP2C1i-3 and PP2C-silenced strains treated with 20 μM PD98059 under cell wall stress (Figure 6A). The measured relative growth rates are illustrated in Figure 6B. In CYM medium, the PP2C1i-1 and PP2C1i-3 strains grew 40% slower than the WT and CK strains, whereas the Slt2i-5 and Slt2i-9 strains grew 50% slower. In addition, the growth of the inhibitor-added strain was 50% slower than that of the WT strain without the inhibitor. In addition, the results showed that, under CFW treatment, the relative growth rates of the Slt2i-5 and Slt2i-9 strains were 7% and 6%, respectively, and the relative growth rates of the PP2C1i-1 and PP2C1i-3 strains were 72% and 73%, respectively. However, the addition of 20 μM PD98059 to the PP2C1i-1 and PP2C1i-3 strains decreased their relative growth by 47% and 44%, respectively, compared with the rates obtained without PD98059 addition. With the addition of 20 μM PD98059 to the WT strain, the relative growth rates were reduced by 50% compared to the WT strain without PD98059. Similarly, after CR stress, the relative growth rates of the Slt2i-5 and Slt2i-9 strains were 18% and 19%, respectively and the relative growth rates of the PP2C1i-1 and PP2C1i-3 strains were 69% and 66%, respectively. The relative growth rates of PP2C1i-1 and PP2C1i-3 treated with 20 μM PD98059 were decreased by 23% and 19%, respectively, compared with those of the untreated PP2C1i-1 and PP2C1i-3 strains. With the addition of 20 μM PD98059 to the WT strain, the relative growth rates were reduced by 35% compared to the WT strain without PD98059. Under SDS treatment, the relative growth rates of the Slt2i-5 and Slt2i-9 strains were reduced by 54%, compared with those of the WT and CK strains. However, the relative growth rates of the PP2C1i-1 and PP2C1i-3 strains were increased by 10% and 12%, respectively, compared with the WT and CK strains. The relative growth rates of both PP2C1i-1 and PP2C1i-3 strains treated with 20 μM PD98059 were decreased by 36% compared with those of the untreated PP2C1i-1 and PP2C1i-3 strains. The addition of 20 μM PD98059 to the WT strain, the relative growth rates were reduced by 40% compared to the WT strain without PD98059. Together, the above-described results demonstrate that Slt2 plays a positive regulatory role in cell wall integrity, whereas PP2C plays a negative regulatory role in cell wall integrity. The addition of a specific inhibitor of Slt2 phosphorylation in the WT was consistent with the effect after silencing of *GlSlt2*, and the addition of a specific inhibitor of Slt2 phosphorylation to PP2C-silenced transformants increased cell wall sensitivity, which indicates that *GlPP2C1* silencing may respond to cell wall integrity by positively regulating Slt2.

## 4. Discussion

At present, edible and medicinal fungus polysaccharides are becoming a research area of intense interest in many disciplines in China and elsewhere due to their unique nutritional value and physiological activity. *Ganoderma lingzhi* is a traditional and vital medicinal mushroom. As it contains GL-PS and other bioactive substances, this mushroom enjoys a high reputation worldwide and is widely used in the food industry. For instance, GL-PS inhibits glioma growth and prolongs the survival of rats by enhancing the cytotoxic activity of natural killer cells and T cells and promoting the functional maturation of dendritic cells [36]. In previous studies, the administration of GL-PS significantly reduced the tumor volume, tumor weight and tumor markers of tumor-bearing mice. Correspondingly, the percentage of relevant immune cells and cytokines in the mice increased, which indicated that GL-PS had an antitumor effect [37]. However, research on the regulatory genes related to GL-PS biosynthesis has rarely been reported. Discovering these regulatory genes will help increase the content of GL-PS in *Ganoderma lingzhi* and help further dissect the regulatory network of polysaccharide synthesis.

Moreover, many studies have shown that GL-PS can protect organisms from harsh environments or pathogens. For example, GL-PS protects fibroblasts from photoaging by eliminating ultraviolet B (UVB)-induced ROS [38]. Heat stress at 42 °C for 2 h significantly changed the metabolism of GL-PS, resulting in an increase of 45.63% in the content of GL-PS in *Ganoderma lucidum*. In addition, the in vitro antioxidant activity of heat-treated polysaccharides is higher than that of untreated polysaccharides, which indicated that GL-PS responds positively to heat stress [39]. The genetic modification of GL-PS is expected to simultaneously improve the biomass and stress resistance of *G. lingzhi*. Therefore, the identification of elite genes that affect GL-PS is critical to improve biomass and stress resistance in *G. lingzhi*. However, to date, the effect of protein phosphatase regulation on GL-PS has not yet been reported. This study provides the first demonstration that regulation of the protein phosphatase *GlPP2C1* plays a negative regulatory role in GL-PS biosynthesis in macrobasidiomycetes and the stress resistance of the *G. lingzhi* cell wall and, thus, provides a reference for further study on the biosynthesis of GL-PS and cell wall stress resistance. This result is similar to that found for PTC1 in yeast. A lack of Ptc1 (a type 2C protein phosphatase) causes hypersensitivity to caffeine, CFW and Congo red or alkalinization of the medium in yeast [40]. In addition, *GlSlt2*, an important protein kinase in the MAPK pathway, also participates in GL-PS biosynthesis and plays a positive regulatory role. Compared with the results found for the WT strain, PP2C silencing increased the GL-PS content and the level of Slt2 phosphorylation, and Slt2 silencing decreased the GL-PS content and the level of Slt2 phosphorylation. Our results also suggested that *GlPP2C1* silencing can increase the content of GL-PS mediated by Slt2. However, whether the change in the GL-PS content is caused by the change in Slt2 phosphorylation needs further study. In short, exploring related regulatory genes based on this line of thinking may be an approach for finding genes related to polysaccharide synthesis. However, both the silencing of *GlPP2C1* or *GlSlt2* and the addition of inhibitors to the WT strain resulted in a slower growth phenotype compared with that of the WT and CK strains in CYM medium. The growth phenotype is affected by multiple complex factors; thus, the slower growth of PP2C- or Slt2-silenced strains may be due to the combined effects of changes in the phosphorylation levels of multiple proteins and cannot be attributed to the effect of changes in the phosphorylation of a particular protein.

**Figure 7:**
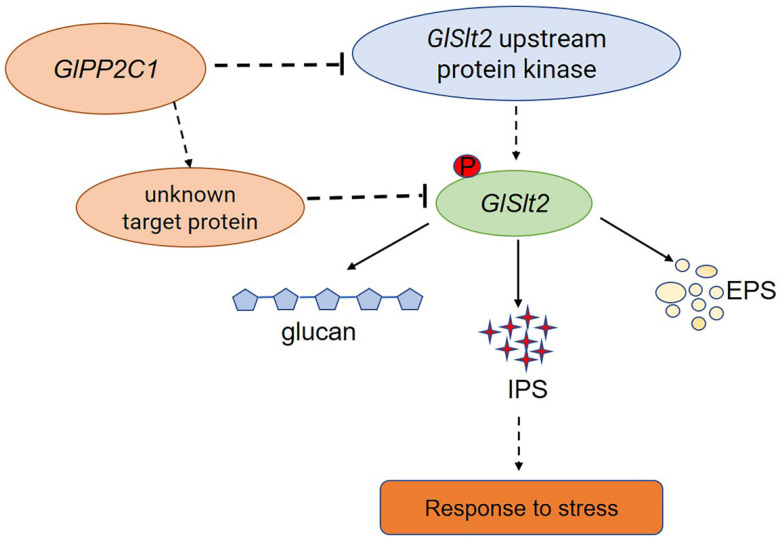
A model of the mechanism through which PP2C negatively regulates GL-PS biosynthesis mediated via Slt2. *GlPP2C1* silencing increases the phosphorylation of Slt2. PP2C may be an upstream protein kinase acting on Slt2 or it may directly act on Slt2 and, thereby, regulate GL-PS (IPS, EPS and β-1,3-D-glucan) biosynthesis in response to cell wall stress.

Research on PP2Cs in fungi has mainly focused on *Saccharomyces cerevisiae*. To date, the published research on PP2Cs has mainly focused on their physiological functions and mechanism of response to the environment. Similar reports have been reported in other species. For instance, a Δptc1 (type 2C protein phosphatases) mutant shows increased tolerance to NaCl, KCl, LiCl and sorbitol and higher sensitivity to caffeine and menadione in *Fusarium oxysporum* [41,42]. Consistent with our results, *GlPP2C1* negatively regulates cell wall integrity stress in *G*. *lingzhi.* The reason for this result may be the phosphorylation level of Slt2 in the PP2C1i-silenced strains. This result was similar to that reported in previous studies [43]. It has been widely reported that the Slt2-MAPK pathway plays an integral role in regulating cell wall repair and integrity. As the activated state of Slt2 is widely believed to occur in parallel with its phosphorylation, the stimulation of this pathway is often easily monitored using commercial antibodies that detect phosphorylated forms of Slt2 [17]. Slt2-specific phosphorylated antibody recognizes PTyr and PThr in a specific sequence context where PThr is the predominant epitope. Our results found that *GlPP2C1* silencing increased the phosphorylation of *GlSlt2*. *GlPP2C1* may regulate the phosphorylation level of Slt2 through two mechanisms: in the first, the increase in phosphorylation is due to *GlPP2C1* acting on the upstream protein kinase of *GlSlt2*, and in the other, *GlPP2C1* indirectly affects *GlSlt2* phosphorylation through key targets. This is because, as well as phosphorylated *GlSlt2*, two unknown phosphoproteins were detected with P-Ser/Thr antibody in the PP2C1-silenced strains (Figure 1C). These results imply that *GlPP2C1* may affect the phosphorylation of *GlSlt2* through these two unknown targets. In any case, further in-depth research is needed to clearly understand the two unknown proteins. The specific regulatory mechanism requires further study (Figure 7). The study of *GlPP2C1* in large basidiomycetes could deepen the understanding of the mechanism of the environmental regulation of growth and development in large basidiomycetes.

To summarize, this study provided insights into the regulation of GL-PS in an essential medicinal mushroom, *Ganoderma lingzhi.* The current research results clearly show that *GlPP2C1* plays a negative regulatory role in GL-PS biosynthesis and that *GlSlt2* plays a positive regulatory role in GL-PS biosynthesis. In addition, we found that *GlPP2C1* silencing increased the phosphorylation of *GlSlt2*. These findings provide an excellent opportunity to determine the potential physiological functions of *GlPP2C1* in *G. lingzhi* and provide a foundation and valuable genetic resources for further study of basidiomycetes.

## Data Availability

Not applicable.

## Supplementary Materials

The following supporting information can be downloaded at: https://www.mdpi.com/article/10.3390/jof8090949/s1, Figures S1–S6: Multiple sequence alignment of PP2C in *G. lingzhi*; Amino acid sequence alignments of the conserved domain in PP2C from four different species; Multiple sequence alignment of PP2C in different species; Structure of the vector constructed for silencing the expression of *GlPP2C1*; Determination of mycelial bifurcation, spacing and biomass in different strains; Different concentrations of PD98059 were added to WT strain. Table S1: Oligonucleotide primers used.
