# Supplementary material for: GlPP2C1 Silencing Increases the Content of Ganoderma lingzhi Polysaccharide (GL-PS) and Enhances Slt2 Phosphorylation
Source: J Fungi (Basel). 2022 Sep 10;8(9):949. doi: 10.3390/jof8090949 (PMC9506439; doi:10.3390/jof8090949)
Supplement: Supplementary file 1 [file jof-08-00949-s001.zip › jof-1841076-supplementary.pdf]

Supplementary materials for

GlPP2C silencing increases the content of *Ganoderma lingzhi* polysaccharide (GL–PS) and enhances Slt2 phosphorylation

Zi Wang, Juhong Chen, Lingshuai Wang, Juan Ding, Mingwen Zhao, Rui Liu*

Affiliations:

1 Key Laboratory of Microbiological Engineering of Agricultural Environment, Ministry of Agriculture; Department of Microbiology, College of Life Sciences, Nanjing Agricultural University, Nanjing 210095, P.R. China

*Corresponding author: Rui Liu

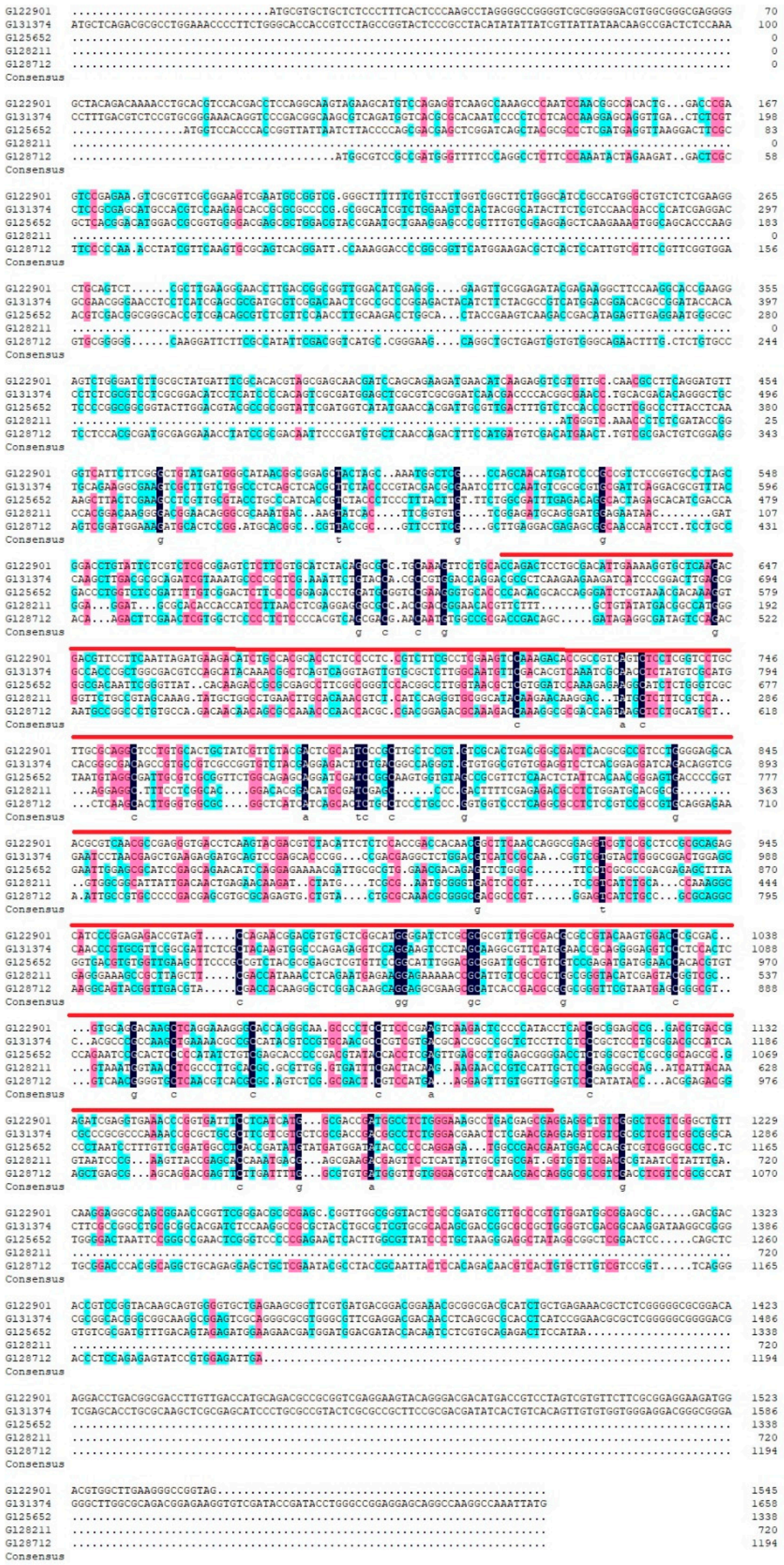
G122901
G131374
G125652
G128211
G128712
Consensus

Figure S1 Multiple sequence alignment of PP2C in *G. lingzhi*. *Gl22901 (OP251201), Gl131374 (OP311594), Gl25652 (OP311595), Gl128211 (OP311596), Gl128712 (OP311597)*. The specificity of the *Gl22901* silenced fragments was demonstrated by multiple sequence alignment of the five PP2Cs of *G. lingzhi* (red lines marked the silenced fragments 614bp–1202bp). Dark blue shading indicated invariant residues, light blue shading and pink shading represent at least 80% conservation and at least 60% conservation, respectively. Nucleotide data comparison was performed on DNAMAN. The alignment was generated with DNAMAN software.

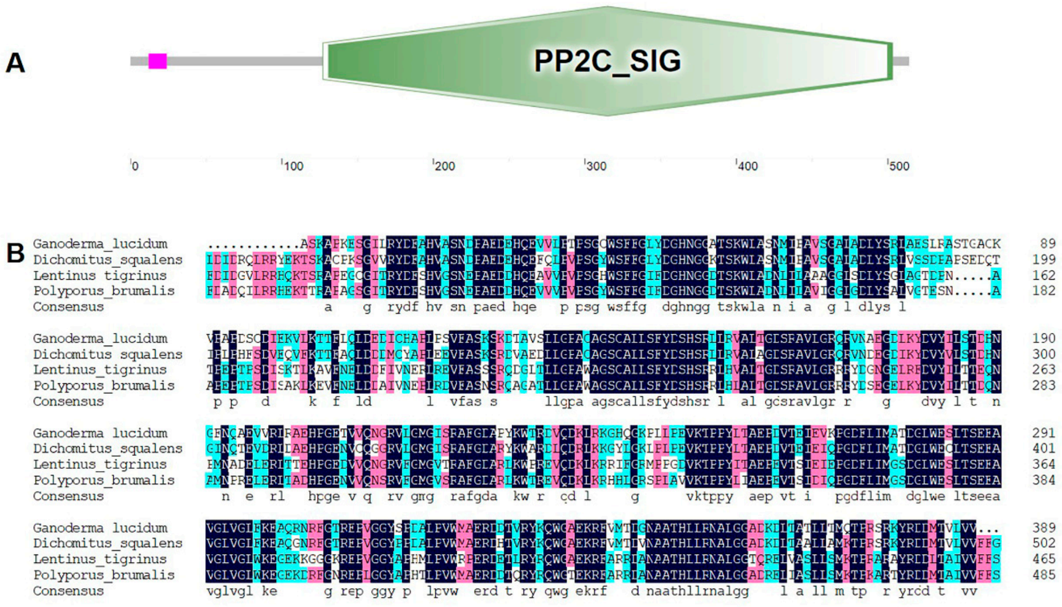

Figure S2 Amino acid sequence alignments of the conserved domain in PP2C from four different species. (A) Sequence analysis of *GlPP2C1*. The pink part indicated a region of low compositional complexity, as predicted by the domain prediction program SMART. The green part indicated a SMART PP2C_SIG domain, as predicted by the domain prediction program SMART. **(B)** Alignment of *GlPP2C1* with other fungal PP2C proteins. The species and GenBank accession numbers were as follows: *G. lingzhi* (GL22901−R1), *Dichomitus squalens* (TBU29298.1), *Lentinus tigrinus* (RPD55288.1), *Polyporus brumalis* (RDX47371.1). Dark blue shading indicated invariant residues, light blue shading and pink shading represent at least 80% conservation and at least 60% conservation, respectively. Protein data comparison was performed on DNAMAN. The alignment was generated with DNAMAN software.

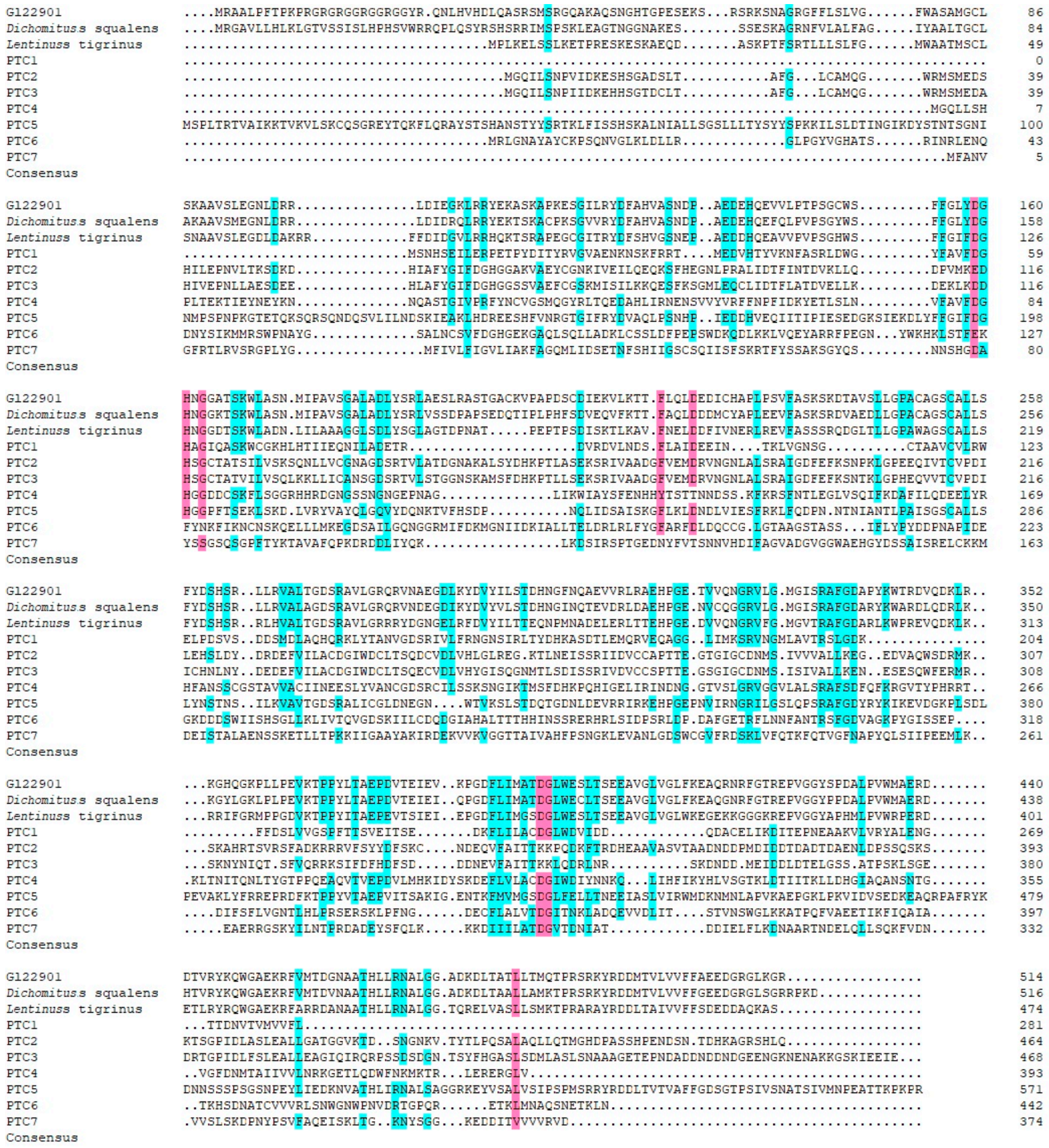

Figure S3 Multiple sequence alignment of PP2C in different species. A multiple sequence alignment of Gl22901 with *D. squalens* (TBU29298.1), *Lentinus tigrinus* (RPD55288.1), *S. cerevisiae*'s PP2C (PTC1 NC_001136.10, PTC2 NC_001137.3, PTC3 NC_001134.8, PTC4 NC_001134.8, PTC5 NC_001147.6, PTC6 NC_001135.5, PTC7 NC_001140.6). Dark blue shading indicated invariant residues, light blue shading and pink shading represent at least 80% conservation and at least 60%

conservation, respectively. Protein data comparison was performed on DNAMAN. The alignment was generated with DNAMAN software.

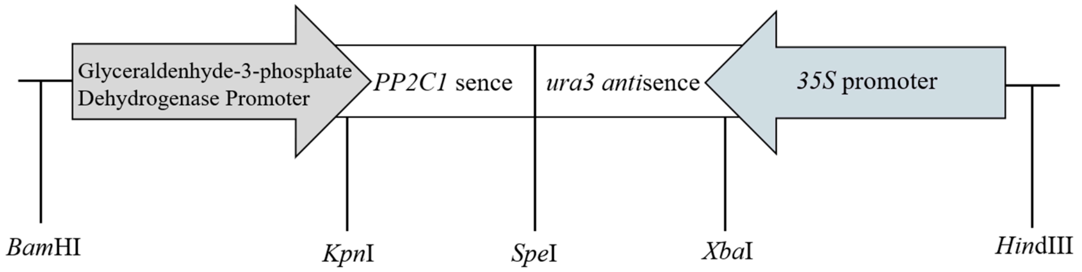

Figure S4 Structure of the vector constructed for silencing the expression of *GlPP2C1*. GPD forward promoter drives *GlPP2C1* gene and ura3 transcription, and 35S reverse promoter drives *GlPP2C1* gene and ura3 transcription. The independent transcription of a target gene from each promoter may produce a pool of sense and antisense RNAs in the cell that combine to form long double-stranded RNA (dsRNAs), which can then be processed into small interfering RNAs (siRNAs) by Dicer to trigger the degradation of homologous mRNAs, which reduces gene expression.

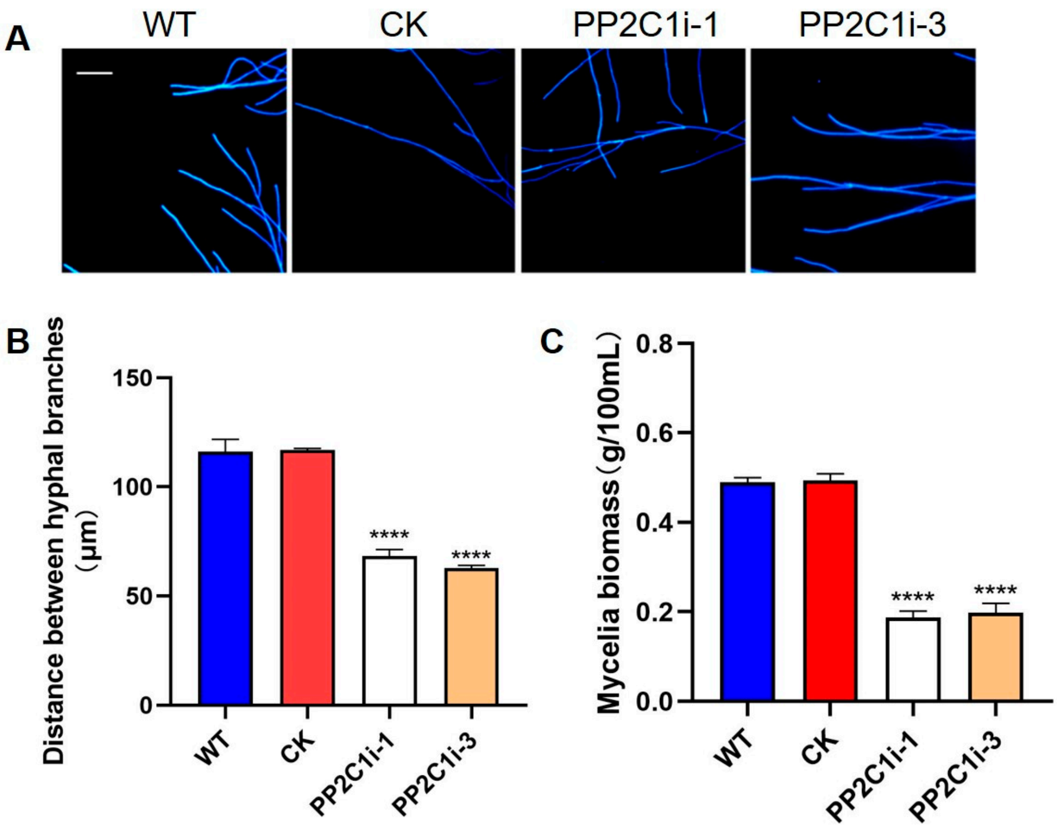

Figure S5 Determination of mycelial bifurcation, spacing and biomass in different strains. **(A)** Fluorescent images of vegetative hyphae suspended in calcofluor white (2.5μg/mL) under a fluorescence microscope. *G. lingzhi* strains were cultivated for 4 days on CYM plates, and vegetative hyphae were stained after they were removed from strongly growing colonies (scale bar = 100μm). **(B)** Distances between hyphal branches of the PP2C1−silenced, WT, and CK strains were measured. **(C)** The biomass of the PP2C1−silenced, WT, and CK strains in liquid culture. The data are presented as the means ± SDs of data from three independent experiments (****P<0.0001 by one−way ANOVA).

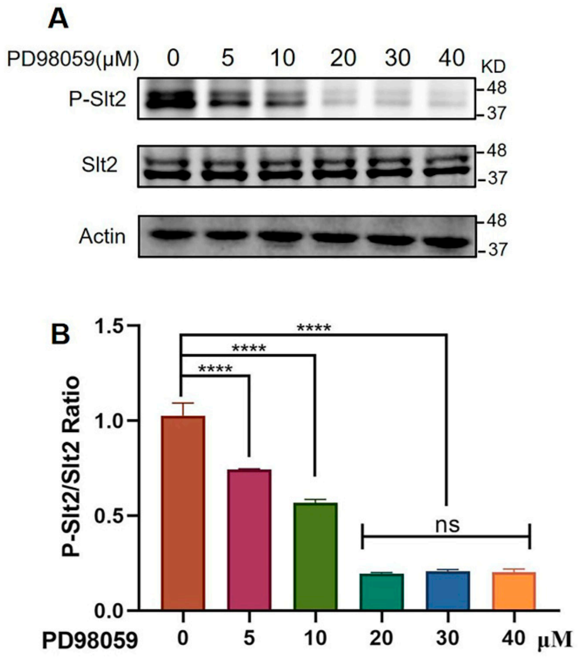

Figure S6 Different concentrations of PD98059 were added to WT strain. (A) Different concentrations of PD98059 are added to WT strain to detect the phosphorylation level of Slt2. **(B)** P-Slt2/Slt2 ratio from panel A. The data are presented as the means ± SDs of data from three independent experiments (****P<0.0001 by one−way ANOVA).

PP2C1 silencing sequence:

CAGACTCCTGCGACATTGAAAAGGTGCTCAAGACGACGTTCCTTCAATTAGATGAGGA
CATCTGCCACGCACCTTTCCCCTCCGTCTTCGCCTCGAAGTCCAAAGACACCGCCGTC
AGTCTCCTCGGTCCTGCTTGCGCAGGCTCCTGTGCACTGCTATCGTTCTACGACTCGCA
TTCCCGCTTGCTCCGTGTCGCACTGACGGGCGACTCACGCGCCGTCCTGGGAGGCAAC
GCGTCAACGCCGAGGGTGACCTCAAGTACGACGTCTACATTCTCTCCACCGACCACAA
CGGCTTCAACCAGGCGGAGGTCGTCCGCCTCCGCGCAGAGCATCCCGGAGAGACCGT
AGTCCAGAACGGACGTGTGCTCGGCATGGGGATCTCGCGCGCGTTTGGCGACGCGCC
GTACAAGTGGACCCGCGACGTGCAGGACAAGCTCAGGAAAGGGCACCAGGGCAAGC
CCCTCCTTCCCGAAGTCAAGACTCCCCCATACCTCACCGCGGAGCCGGACGTGACCGA
GATCGAGGTGAAACCCGGTGATTTCCTCATCATGGCGACCGATGGCCTCTGGGAAAGC
CTGACGAGCGA

Table S1. Oligonucleotide primers used

Primer	Sequence (5′to 3′)	Description
PP2C1−pmi−F	GGGGTACCCAGACTCCTGCGACATTG	Used to get the silencing fragment of the *PP2C1* gene
PP2C1−pmi−R	GACTAGTTCGCTCGTCAGGCTTT	
RT−PP2C1−F	GGGAAAGCCTGACGAGCGA	Used to detect the *PP2C1* expression
RT−PP2C1−R	GCCATCCACACGGGCAACG	
RT−18S−F	TCGAGTTCTGACTGGGTTGT	Used to detect the 18S rRNA expression
RT−18S−R	TCCGTTGCTGAAAGTTGTAT	
